# Temperature alters the inotropic and chronotropic effect of D_1_-dopamine receptor stimulation in the mammalian atrium

**DOI:** 10.1007/s00210-025-04313-6

**Published:** 2025-05-30

**Authors:** Peter Grundig, Thanh Hoai Pham, Britt Hofmann, Joachim Neumann, Ulrich Gergs

**Affiliations:** 1 Institute for Pharmacology and Toxicology, Medical Faculty, Martin-Luther-University Halle-Wittenberg, Magdeburger Straße 4, 06097 Halle (Saale), Germany; 2 Department of Cardiac Surgery, Mid-German Heart Centre, University Hospital Halle, Ernst-Grube-Str. 40, D‑06097 Halle (Saale), Germany

**Keywords:** Dopamine, D_1_-dopamine receptor, Hypothermia, Hyperthermia, Arrhythmia, Inotropy, Chronotropy

## Abstract

We studied how hypothermia and hyperthermia may change the efficacy and/or potency of dopamine to augment the force of contraction in atria of mice with cardiac overexpression of the human D_1_-dopamine receptor (D_1_-TG). We measured the force of contraction on paced (1 Hz) left atria and spontaneously beating right atria of these D_1_-TG mice in vitro. The intrinsic heart beat in the right atria from D_1_-TG mice lowered when we reduced the temperature (24 °C, hypothermia) and elevated when we raised the temperature (42 °C, hyperthermia) in the organ baths. In addition, the efficacy of dopamine (0.001–100 µM) to augment the force of contraction was diminished in the left and right D_1_-TG mouse atrial preparations under 24 °C and 42 °C compared to 37 °C in the organ baths. Likewise, the rise in force after dopamine was diminished at 24 °C and 42 °C compared to 37 °C in paced human atrial preparations (HAP) obtained from patients who underwent surgery. In conclusion, the inotropic effects of dopamine in D_1_-TG mice and in human atrial preparation via D_1_-dopamine receptors, but also the effects of dopamine in D_1_-TG mice on the heartbeat, change with ambient temperature.

## Introduction

In patients, dopamine causes an increase in force of contraction and a rise in the heart beat. Concomitantly, dopamine can augment the velocity which force augments and declines, but dopamine also shortens the time the heart needs to relax (review: Neumann et al. 2023). These effects are mediated by different G-protein coupled receptors: the β-adrenoceptor, the α-adrenoceptor and the D_1_-dopamine receptor (Neumann et al. 2023, Gergs et al. 2024).

**Fig. 1:**
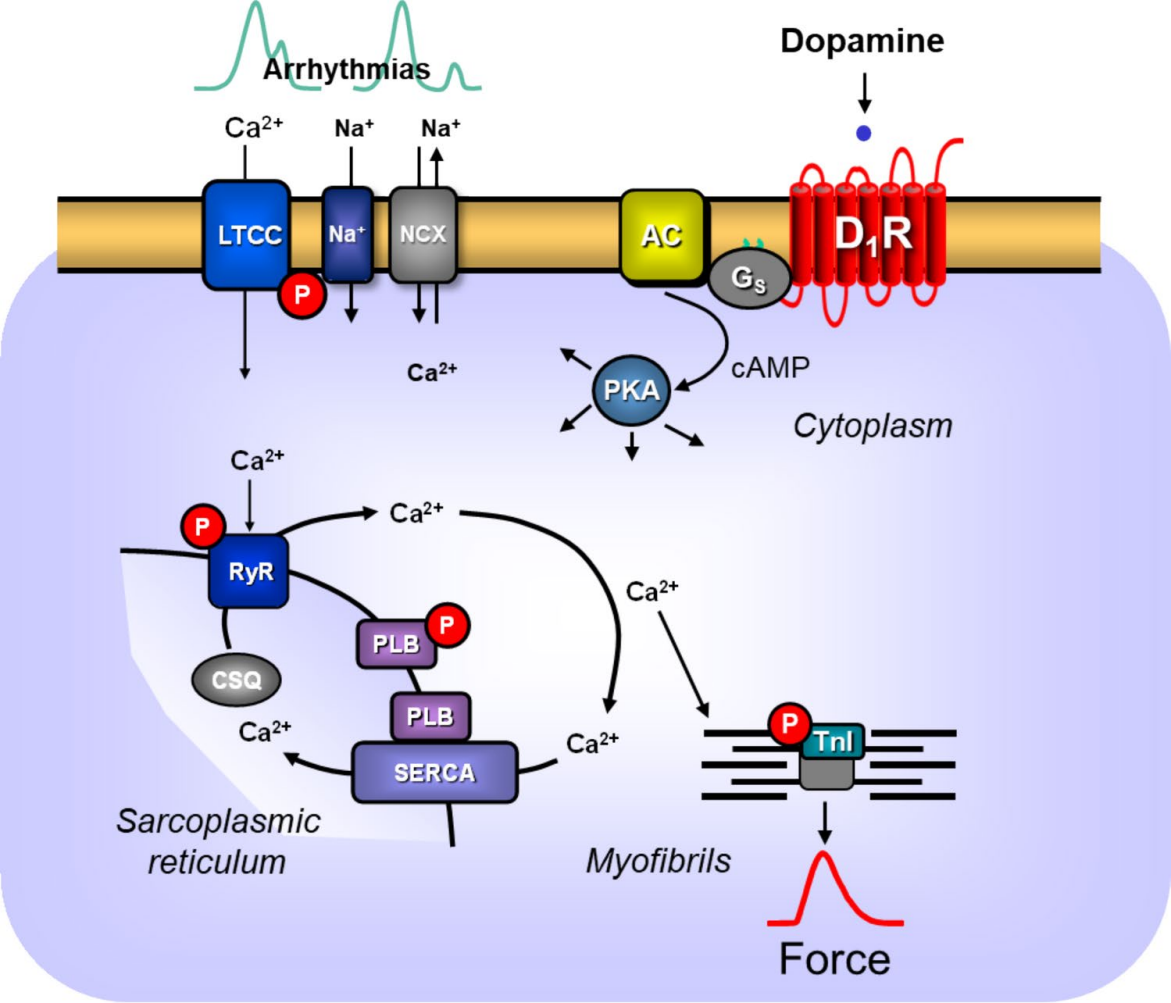
Scheme for the signal transduction of cardiac D_1_-dopamine receptors. Dopamine can stimulate D_1_-dopamine receptors (D_1_R) in the D_1_-TG mice. Then, stimulatory GTP binding proteins (G_s_) contribute to more production of cAMP catalyzed by adenylyl cyclase (AC) located at the sarcolemma. This cAMP will bind to and activate the protein kinase A (PKA) that is typically activated by phosphorylation of regulatory proteins. A higher phosphorylation state of the inhibitory subunit of troponin (TnI) probably causes a faster relaxation of the heart. An enhanced phosphorylation state of the L-type Ca^2+^ current (LTCC) causes an increase in the Ca^2+^ inflow into the cell. This Ca^2+^ then releases Ca^2+^ through the ryanodine receptor (RyR in the sarcoplasmic reticulum). Phosphorylation of RyR increases the release of Ca^2+^ from the sarcoplasmic reticulum and thus hastens the time to peak tension. Phosphorylation of phospholamban (PLB) which activates SERCA and thereby accelerates the removal of Ca^2+^ from the cytosol, results in hastened mechanical relaxation. When more sodium ions pass through the sarcolemma via a sodium channel (Na +), the intracellular sodium ions will rise: they can now leave the cell via the sodium calcium exchanger NCX. As a result of the electrogenic transport through the NCX, the cardiomyocytes may depolarize, and as a direct consequence, early and late afterdepolarization could occur, leading to cardiac arrhythmias

In a recent study, we generated and studied in some detail transgenic mice (D_1_-TG) with cardiac overexpression of the human D_1_-dopamine receptor (Rayo Abella et al. 2024). D_1_-TG responded to dopamine (in the presence of propranolol) with positive inotropic effect (PIE) and positive chronotropic effect (PCE) in the organ bath (Rayo Abella et al. 2024), and dopamine augmented the phosphorylation state of phospholamban (PLB) (Rayo Abella et al. 2024) (Scheme: Fig. 1). Similarly, dopamine could increase FOC via D_1_-dopamine receptors in HAP (Gergs et al. 2024).

The present study set out to study the dichotomic functions of human D_1_-dopamine receptors at low and high temperatures in the heart. Low and high ambient temperatures can change the mechanical function of human atrial or ventricular preparations (Fig. 1) (review: Mackiewicz and Lewartowski 2006, Antzelevitch and Yan 2010, Fukaya et al. 2017, Hiis et al. 2018). On the one hand, hypothermia is sometimes used in the clinic to reduce the metabolic demand of patients during a time-demanding surgery on the heart or on the brain (Choi et al. 2023). In some cases in the Northern Hemisphere in winter, sailors, soldiers, mountaineers and skiers are exposed to cold wind and/or cold water (Jones et al. 2023, Bennett and Holcomb 2017, Procter et al. 2018). Hypothermia can induce deadly arrhythmias in such persons (Dietrichs et al. 2019). Hypothermia is sometimes used to study the causes of arrhythmias more specifically Osborn waves, clinically and in the organ bath (Antzelevitch and Jan 2010, Kloesel et al. 2011, Eroglu et al. 2019, Topscu et al. 2019).

On the other hand, hyperthermia is sometimes used in the clinic, for instance, during melanoma surgery in order to reduce the tumour size by impairing proliferation of tumour cells (Shidnia et al. 1990). So-called malignant hyperthermia can be caused by mutations of the ryanodine receptors (Fig. 1) in patients or by drugs like thyroid hormones, neuroleptic drugs or anaesthetic drugs and muscle relaxant drugs in the clinic (Burger and Philippe 1992, Gregory and Weant 2021). Malignant hyperthermia can precipitate cardiac arrhythmias (Chakraborty et al. 2023, Sun et al. 2021). Fever, in the course of sepsis, e.g. in the course of a COVID infection, can cause cardiac hyperthermia (Chen et al. 2023; Santoro et al. 2022).

Moreover, dopamine is used clinically. Dopamine is often applied via intravenous lines, e.g. in the intensive care unit, to increase cardiac beating rate and the cardiac output (Neumann et al. 2023). Sometimes dopamine is infused in patients with poor arterial perfusion of the kidney to induce vasodilation of the renal artery and thus improve the function of the kidney (Russel et al. 2021, Joannidis et al. 2017). D_1_-dopamine receptors are stimulated by fenoldopam (Gergs et al. 2024). Fenoldopam is sometimes used to treat hypertension and to induce controlled hypotension (Zhou et al. 2021): this is thought mainly to be due to dilation of arterial resistance vessels (Degoute 2007). Moreover, fenoldopam has been reported to be beneficial in some patient groups with heart failure, possibly via direct stimulation of D_1_-dopamine receptors on cardiomyocytes (Young et al. 1988). We have presented functional evidence that fenoldopam can stimulate D_1_-dopamine receptor in isolated human atrial preparations, and this led to a positive inotropic effect (Gergs et al. 2024). Others presented evidence that the D_1_-dopamine receptor is present in human ventricular cardiomyocytes and that its expression increases in human heart failure (Yamaguchi et al. 2020).

Please consider that similarities exist between the effects of serotonin and histamine and those of dopamine in the human heart. Like dopamine, serotonin and histamine can be produced in the heart of humans (Gergs et al. 2017, Neumann et al. 2021). Serotonin and histamine make the human heart beat stronger and quicker. Serotonin and histamine increase the cyclic adenosine monophosphate (cAMP) concentrations and the phosphorylation state of phospholamban in the heart of man. Like dopamine, serotonin or histamine does not augment the force of contraction in wild-type mouse atrial preparations (Gergs et al. 2019a, Gergs et al. 2021, Rayo Abella et al. 2024). Hearts from mice with overexpression of the human 5-HT_4_-serotonin receptor (5-HT_4_-TG) or with overexpression of the human H_2_-receptor (H_2_-TG) respond to serotonin or histamine with increased force generation and therefore mimic the functions of the human heart in this respect (Gergs et al. 2010, Gergs et al. 2019a). Arrhythmias appeared with increased incidence in right atrial preparations from 5-HT_4_-TG at low temperatures in the organ bath than in their WT littermates (Gergs et al. 2021). In addition, the potency of serotonin was enhanced, whereas the efficacy of serotonin or histamine was reduced, in isolated left atria from 5-HT_4_-TG under low temperatures in the organ bath (Gergs et al. 2021; Hoffmann et al. 2023). Hence, we studied here whether or not dopamine exerts similar inotropic and proarrhythmic effects as serotonin or histamine in a suitable mouse model. Moreover, to find out whether these findings in transgenic mice may have any clinical relevance, we finally studied HAP.

In sum, we addressed the following assumptions: (i) hypothermia and hyperthermia in comparison with normothermia change atrial contractility after stimulation with dopamine in humans and in D_1_-TG mice via D_1_-dopamine receptors and (ii) hypothermia and hyperthermia in comparison with normothermia augment the incidence of atrial arrhythmias in D_1_-TG mice. Parts of this work have been reported in an abstract form (Grundig et al. 2024).

## Materials and methods

### Transgenic mice

We have already established the transgenic mice (i.e. D_1_-TG mice) in a previous study (Rayo Abella et al. 2024). D_1_-TG mice show a cardiac-specific overexpression of the human D_1_-receptor as a consequence of the use of the α-myosin heavy chain promoter (Rayo Abella et al. 2024). We studied both sexes, and the age was around 280 days. We treated and grew the animals with permission from the Animal Welfare Committee of the Martin-Luther-University of Halle-Wittenberg, Halle, Germany (TS 10–24). Solely in D_1_-TG, but not in WT, dopamine exerts a positive inotropic effect (Rayo Abella et al. 2024). We measured unchanged endogenous and augmented levels of human D_1_-receptors by polymerase chain reaction (PCR) in hearts from D_1_-TG compared to hearts from WT (Rayo Abella et al. 2024). We detected D_1_-receptors in D_1_-TG in Western blots and by employing autoradiography in atria from D_1_-TG (Rayo Abella et al. 2024).

### Contractility in isolated atria from mouse and human

**Fig. 2:**
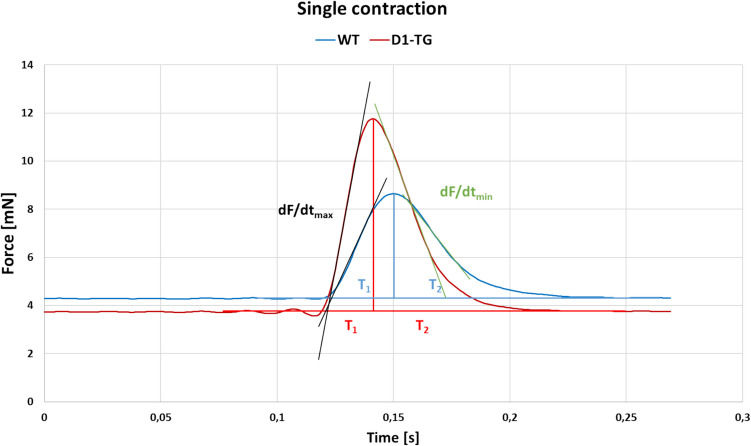
Measurements on a single contraction of WT and D_1_-TG mice. Typical original recording of a single contraction of WT and D_1_-TG mice after 300 nM dopamine application. The baseline and peak line were placed by the software LabChart 8 under the curves to plot time to peak tension (T_1_) and time to relaxation (T_2_) (blue lines for WT and red lines for D_1_-TG mouse). The tangents on the curves represent the maximum positive (dF/dt_max_, black tangents) and negative rate of tension development (dF/dt_min_, green tangents). Ordinate depicts developed force of contraction in millinewton (mN). Abscissa shows time in seconds (s)

Hearts were cut and atria were separated from the other cardiac tissue under a dissecting microscope. Atria were vertically mounted in commercially available double-barrelled organ baths (Willers, Münster, Germany: Neumann et al. 2003, Gergs et al. 2013, Gergs et al. 2021). We stimulated mouse left atrial preparations and human atrial preparations with rectangular impulses for 5 ms at 1 Hz (Grass Stimulator SD 9, Quincy, MA, USA). The voltage amounted to about 10% over the minimum voltage to start the atria contracting. To start the beating in left atrial preparations of mice, this voltage is usually 5 V. The temperature of the buffer in the organ bath was at the start of the experiment 37 °C using a thermostat (Neumann et al. 1998, 2003, Gergs et al. 2021; Hoffmann et al. 2023). The force was measured under isometric conditions using a Hellige (Freiburg, Germany) force transducer, a bridge amplifier (ADInstruments) and digitized (PowerLab, AD Instruments), fed into a Dell personal computer and quantified with respect to force, its first derivative, time to peak force, time of relaxation, and beating rate using a commercial software (GraphPad 9 from ADInstruments). An example of the measurements on a single contraction is shown in Fig. 2 for WT and D_1_-TG mice after dopamine application at 37 °C. We thought it was important to report both the force of contraction and its first derivative (rate of tension development and rate of relaxation) because others noted that in human ventricular muscles, the force increased while the rate of tension development declined. At low temperatures, the muscle reached its higher force a much later time than at normothermia (Hiis et al. 2018). Hence, providing the first derivative of force versus time can reveal otherwise overlooked insights.

When force had completely stabilized (which was electrically stimulated at 1 Hz for the murine or human atria, the cardiac temperature we decreased temperature in the organ baths by direction of the pumps from heated water to a second reservoir of precooled water. More details can be found elsewhere (Gergs et al. 2021). When the low temperature experiment had been completed, the temperature in the organ bath was returned to 37 °C, and using a thermostat, a temperature of 42 °C was achieved in the organ bath. Having finished the experiment at high temperature, the temperature was again lowered to 37 °C; a cumulative concentration–response curve to dopamine was constructed. Dopamine was then washed out, temperature was lowered and a further concentration–response curve to dopamine was performed. Thereafter, dopamine was washed out after we had switched again to 37 °C. Subsequently, hyperthermia was induced and a third concentration–response curve to dopamine was constructed. The samples were again returned to normothermia and washed out. We have several organ baths with thermostats in our department. Thus, we can study always on the same experimental day the same number of atrial preparations from WT and D_1_-TG. Usually, we randomized such that left atria and right atria were mounted in alternative organ baths. We use the same reagents on the same experimental day for all organ baths to minimize differences between genotypes. The temperature is changed in all organ baths at the same time. We check the temperature in parallel with an electric thermometer to ascertain that the intended temperatures are reached.

We decided to study contraction at hypothermia of 24 °C to facilitate comparison with our own previous studies (Gergs et al. 2021; Hoffmann et al. 2023). Moreover, others often used this or a similar hypothermia for their own contraction studies in mammalian heart preparations (e.g. 25 °C: rabbit ventricular cardiomyocytes: Puglisi et al. 1996, Puglisi et al. 1999, guinea pig atria: Tenner and McNeill 1978; 24 °C: guinea pig atria and rat papillary muscles: Miyamoto et al. 2001, rat right papillary muscle: Dobrunz and Berman 1994, 21–23 °C: adult rat ventricular cardiomyocytes: Backx and Ter Keurs 1993, 22.5 °C: mouse right ventricular free wall: Stull et al. 2006). Others argue that in many prior physiological and electrophysiological studies, the investigator worked at “room temperature” which may be approximated to 22.5 °C (Stull et al. 2006). In ground squirrels, cardiac contractions could still be measured at 7 °C, but that seems to be peculiar to mammalian hibernators and not to humans (Nakipova et al. 2017). So when we use 24 °C, we are using even a higher temperature than some previous researchers.

Likewise, we chose to use 42 °C as a model of hyperthermia, because we have used this before (Gergs et al. 2021; Hoffmann et al. 2023) and because others used this or similar temperatures. For instance, whole animals were treated for 15 min with 42 °C, or muscle strips were treated in the organ bath with 42 °C, and then heart function was studied (mouse: Xi et al. 1998, rat: Currie et al. 1988, guinea pig: Reinhardt et al. 1978).

We performed the contractile studies HAP with the same equipment as in the preceding paragraph. The samples were obtained from two male patients and three female patients, aged from 64 to 82 years. Cardiac morbidities were severe coronary heart disease, hypertension, heart failure, and atrial fibrillation. Drug therapy included acetylsalicylic acid, apixaban, hydrochlorothiazide and bisoprolol. Our methods used for human atrial muscle strips have already been reported (Gergs et al. 2009, 2017). In principle, drug application was like in the preceding paragraph. All patients gave written informed consent. The study was approved by the local ethics committee (permit #hm-bü).

In this study, we decided to form two classes of arrhythmias, namely benign and malignant arrhythmias. We define malignant arrhythmias as arrhythmias that are not benign arrhythmias. We define benign arrhythmias as those where only the amplitude of the contraction varies from beat to beat or where the time between beats is different or a single extrasystole is seen.

### Data analysis

We present mean values ± standard error of the mean in the figure. We estimated the statistical significance of differences or comparisons between groups with the help of an analysis of variance (ANOVA), and we then performed the Bonferroni *t*-test or we chose the chi-squared (${\chi }^{2}$) test or paired *t*-tests, as delineated in the figure legends. We defined a *p*-value smaller than 0.05 as significant. Experimental data on positive agonist-induced inotropic and chronotropic effects were studied by fitting sigmoidal curves to the experimental data using GraphPad Prism 5.0 (GraphPad Software, San Diego, CA, USA). All other statistical analyses were calculated as given in the presented figures and tables, which were also computed using GraphPad Prism 5.0.

### Drugs and materials

Dopamine hydrochloride and propranolol hydrochloride were purchased from Sigma-Aldrich (now Merck), Dreieich, Germany. All other chemicals were of the highest purity grade commercially available. Deionized water was used throughout the experiments. Stock solutions were prepared fresh daily.

## Results

### Mouse atrial preparations

**Fig. 3:**
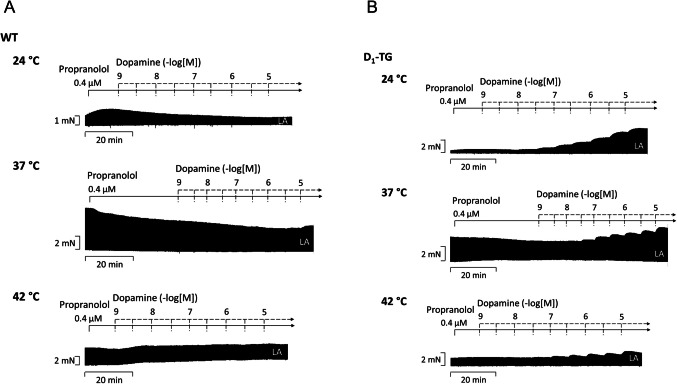

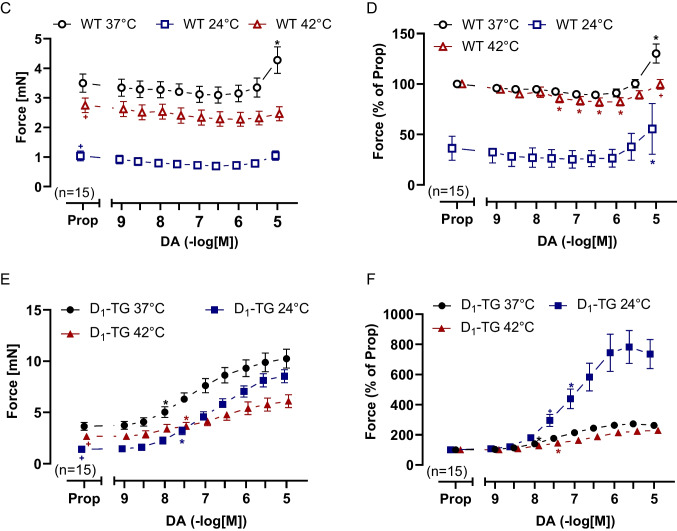
Dopamine increases force of contraction in D_1_-TG but not in WT mice. Original recordings of the effect of increasing concentrations of dopamine on electrically stimulated left atrial (LA) preparations from WT (**A**) or D_1_-TG mice (**B**) under normothermia (37 °C), hypothermia (24 °C) and hyperthermia (42 °C). In addition, the concentration-dependent effects of dopamine on force of contraction in LA were summarized for WT in absolute values (**C**) and in percent % (**D**) or for D_1_-TG in absolute values (**E**) and in % (**F**). In samples, we added 0.4 µM propranolol to the organ bath in order to block β-adrenoceptors. Ordinates in **A**, **B**, **C**, and **E** depict developed force of contraction in millinewton (mN). Abscissae indicate concentrations of dopamine in negative decadic molar concentrations. Temperatures of the organ baths are indicated as circles (37 °C), squares (24 °C), or triangles (42 °C). “n” indicates the numbers of experiments.* first *p <* 0.05 vs. Prop, ^+^ first *p <* 0.05 vs. 37 °C (ANOVA Bonferroni)

Typical original tracings on the time-dependent and concentration-dependent PIE of dopamine are presented in Fig. 3A for WT and in Fig. 3B for D_1_-TG. From a comparison of Fig. 3A and B, it becomes obvious that dopamine only increased force in LA from D_1_-TG but not from WT, which is also statistically evaluated in Fig. 3C and D for WT and in Fig. 3E and F for D_1_-TG mice. Moreover, the PIE of dopamine is observed earlier (i.e., at lower concentrations of dopamine) in the D_1_-TG at 37 °C and 24 °C than at 42 °C in LA. In addition, in the D_1_-TG mouse left atrial preparations, the maximum positive inotropic effects were larger during normothermia than during hyperthermia and hypothermia (Fig. 3E and F in percentage). The potencies for dopamine to augment force in LA from D_1_-TG are compared in Table 1. The potencies of dopamine were significantly lower in hypothermia and hyperthermia than at normothermia (Table 1). Basal tension was less in hypothermia and hyperthermia than at normothermia (numerical values in Table 2).

**Table 1:** Comparison of half-maximal (EC_50_-values) positive inotropic and chronotropic effects of dopamine in isolated left atrial preparations (to assess the contraction force) and right atrial preparations (to assess the beating rate) from D_1_-TG. Negative decadic logarithms of molar drug concentrations are plotted. ^+^*p <* 0.05 vs. 37 °C

Temperature	Left atrial force	Frequency	Right atrial force
37 °C	5.4 ± 0.1 (*n* = 15)	6.1 ± 0.2 (*n* = 12)	1.7 ± 0.3 (n = 15)
24 °C	17.1 ± 0.1^+^ (*n* = 15)	16.2 ± 0.2^+^ (*n* = 14)	1.4 ± 0.2 (n = 15)
42 °C	18.9 ± 0.7^+^ (*n* = 15)	0.1 ± 0.1^+^ (*n* = 9)	5.8 ± 0.1^+^ (*n* = 15)

**Table 2:** Basal parameters of the human and mouse atrial preparations from D_1_-TG. ^+^*p <* 0.05 vs. 37 °C

	**37 °C**	**24 °C**	**42 °C**
**Human right atrium**			
Basal tension [mN]	4.27 ± 1.49 (*n* = 6)	2.48 ± 1.07^+^ (*n* = 4)	4.14 ± 0.58^+^ (*n* = 6)
Basal time-to-peak tension [ms]	71.91 ± 23.78 (*n* = 6)	99.07 ± 7.23 (*n* = 4)	64.35 ± 12.2 (*n* = 6)
Basal relaxation time [ms]	194.13 ± 14.11 (*n* = 6)	274.3 ± 63.2 (*n* = 4)	160.03 ± 25.76 (*n* = 6)
**Mouse left atrium**			
Basal tension [mN]	3.64 ± 0.37 (*n* = 15)	1.41 ± 0.26^+^ (*n* = 15)	2.66 ± 0.27^+^ (*n* = 15)
Basal time-to-peak tension [ms]	17.65 ± 1.95 (*n* = 15)	33.52 ± 1.7^+^ (*n* = 15)	15.12 ± 2.01^+^ (*n* = 15)
Basal relaxation time [ms]	35.05 ± 2.04 (*n* = 15)	59.61 ± 4.32^+^ (*n* = 15)	22.14 ± 1.37^+^ (*n* = 15)
**Mouse right atrium**			
Basal tension [mN]	1.99 ± 0.33 (*n* = 15)	1.12 ± 0.28 (*n* = 15)	1.27 ± 0.23 (*n* = 15)
Basal time-to-peak tension [ms]	15.16 ± 0.92 (*n* = 15)	27.01 ± 4.29^+^ (*n* = 15)	14.32 ± 0.87 (*n* = 15)
Basal relaxation time [ms]	28.38 ± 1.55 (n = 15)	45.38 ± 4.1^+^ (*n* = 15)	20.43 ± 0.79^+^ (*n* = 15)
Basal beating rate [bpm]	214.52 ± 15.58 (*n* = 12)	59.96 ± 4.54^+^ (*n* = 14)	267.82 ± 30.72 (*n* = 9)

**Fig. 4:**
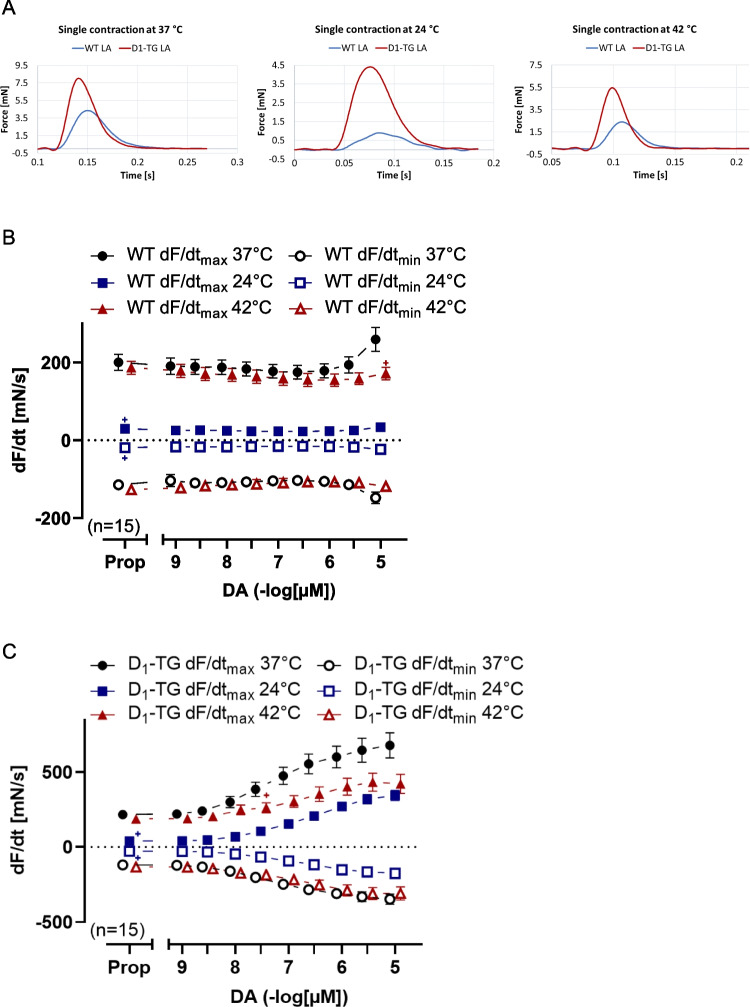
Dopamine increases the rate of tension development and relaxation in left atrium in D_1_-TG. Original recordings of single contractions after 300 nM dopamine application (Fig. 4 A) and concentration response curves for dopamine in electrically stimulated mouse LA from WT (Fig. 4B) and D_1_-TG mice (Fig. 4 C) under normothermia (37 °C), hypothermia (24 °C) and hyperthermia (42 °C). We added 0.4 µM propranolol to the organ bath in order to block β-adrenoceptors. Dopamine induced an increase in the rate of tension development and relaxation in D_1_-TG mice under all thermal conditions. The ordinate in **A** depicts the developed force of contraction in millinewton (mN), and the ordinates in **B** and **C** depict the rate of tension development (dF/dt_max_) and the rate of tension relaxation (dF/dt_min_) in millinewton per second (mN/s). The abscissa in **A** shows time in seconds (s), whereas the abscissae in **B** and **C** indicate concentrations of dopamine in negative decadic logarithm of molar concentrations. Temperatures of the organ baths are indicated as circles (37 °C), squares (24 °C), or triangles with tip up (42 °C). “n” indicates the numbers of experiments. * first *p <* 0.05 vs. Prop, ^+^ first *p <* 0.05 vs. 37 °C (ANOVA Bonferroni)

The effects of 300 nM dopamine on a single contraction in left atrial preparations from WT and D_1_-TG mice at normo-, hypo- and hyperthermia are shown in Fig. 4A. Compared to the WT slope, it is apparent that the slope from D_1_-TG mice is steeper under all temperature conditions. Dopamine in D_1_-TG mice raised the maximum positive and the maximum negative rate of tension development (Fig. 4C). Oppositely, dopamine did not augment the rate of tension development or the rate of relaxation in WT mice under hypo- and hyperthermia (Fig. 4B). Furthermore, dopamine was less effective at raising the maximum positive rate of tension development in D_1_-TG mice under hypothermia and hyperthermia than under normothermia (Fig. 4C). In contrast, the maximum negative rates of tension relaxation increased in absolute terms to similar values by dopamine at normothermia as in hyperthermia but increased less in hypothermia (Fig. 4C).

**Fig. 5:**
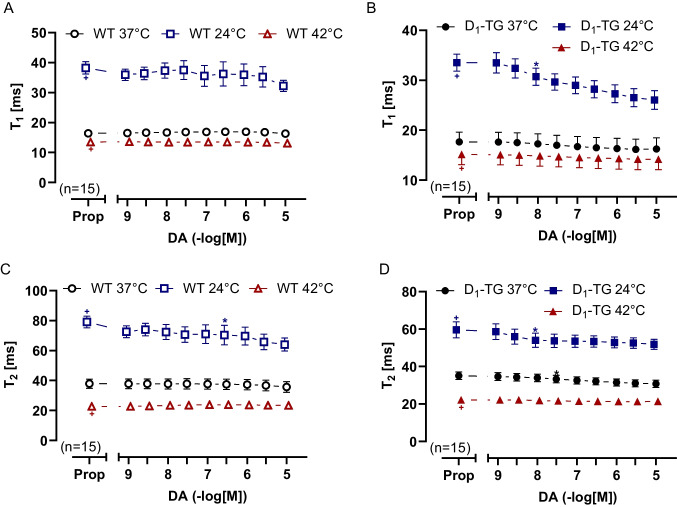
Dopamine shortens time to peak tension and relaxation in left atrium. Time to peak tension curve of WT (**A**) or D_1_-TG (**B**) and time to relaxation curve of WT (**C**) or D_1_-TG (**D**) under normothermia (37 °C), hypothermia (24 °C) and hyperthermia (42 °C). We also added 0.4 µM propranolol to the organ bath in order to block β-adrenoceptors. Time to peak tension and time to relaxation under basal conditions (Prop) were longer under hypothermia than under normothermia or hyperthermia in both LA from WT (**A** and **C**) or D_1_-TG (**B** and **D**). In addition, dopamine shortens time to peak tension in D_1_-TG mice only under hypothermia and shortens time to relaxation under hypothermia and normothermia. Ordinates in **A** and **B** depict time to peak tension (T_1_) and in **C** and **D** time to relaxation (T_2_) in milliseconds (ms). Abscissae indicate concentrations of dopamine in negative decadic logarithm of molar concentrations. Temperatures of the organ baths are indicated as circles (37 °C), squares (24 °C), or triangles with tip up (42 °C). “n” indicates the numbers of experiments. * first *p <* 0.05 vs. Prop, ^+^ first *p <* 0.05 vs. 37 °C (ANOVA Bonferroni)

The time to peak tension under control conditions (Prop) was elevated during hypothermia compared to normothermia or hyperthermia in both LA from WT or D_1_-TG (Fig. 5A and B). Similarly, results are seen in Fig. 5C and D (Table 2), in agreement with findings in the human samples in our previous study (Gergs et al. 2021). Dopamine reduced the time to peak tension at hypothermia only in LA from D_1_-TG (Fig. 5B). Similarly, dopamine shortened the time of relaxation in LA from D_1_-TG at hypothermia and normothermia (Fig. 5D), but also in LA from WT at hypothermia only (Fig. 5C), which can be also seen in Fig. 4A.

**Fig. 6:**
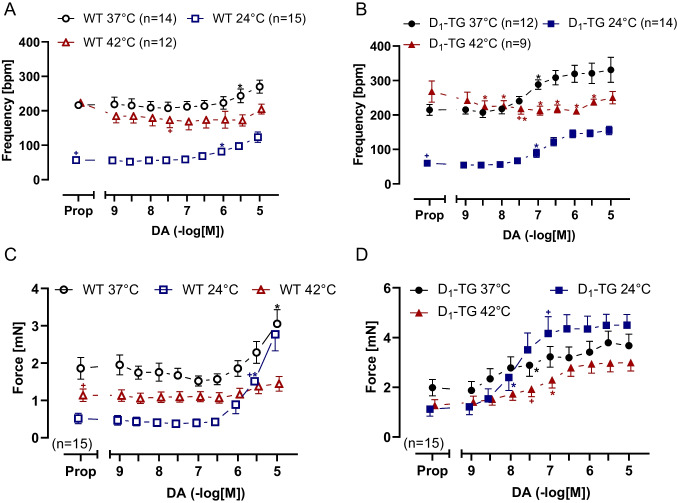
Dopamine increases the beating rate and the force of contraction in the right atrium. Summarized concentration response curves for the effect of dopamine on the beating rate in spontaneously beating mouse right atrial (RA) preparations under normothermia (37 °C), hypothermia (24 °C) and hyperthermia (42 °C) in WT (**A**) or D_1_-TG mice (**B**) and on the force of contraction in millinewton (mN) in WT (**C**) or D_1_-TG mice (**D**). We also added 0.4 µM propranolol to the organ bath in order to block β-adrenoceptors. Ordinates in **A** and **B** depict beats per minute (bpm), ordinates in **C** and **D** depict developed force of contraction in millinewton (mN). Abscissae indicate concentrations of dopamine in negative decadic logarithm of molar concentrations. Temperatures of the organ baths are indicated as circles (37 °C), squares (24 °C), or triangles with tips up (42 °C). “n” indicates the numbers of experiments. * first *p <* 0.05 vs. Prop, ^+^ first *p <* 0.05 vs. 37 °C (ANOVA Bonferroni)

Under basal conditions, the beating rate from D_1_-TG was lower during hypothermia than during normothermia and higher during hyperthermia until 10 nM dopamine administration, compared to normothermic conditions (Fig. 6B, Table 2). The data are statistically evaluated in Fig. 6A for WT and Fig. 6B for D_1_-TG mice, which show that dopamine, starting at 10 nM, potently increased the beating rate in RA from D_1_-TG under normo- and hypothermia (Fig. 6B), while in RA from WT (Fig. 6A), higher dopamine concentrations are needed to increase the beating rate. Dopamine was less potent to raise the beating rate at hypothermia than at normothermia (Table 1). Basal beating rate was lower at hypothermia than at normothermia (Table 2).

**Fig. 7:**
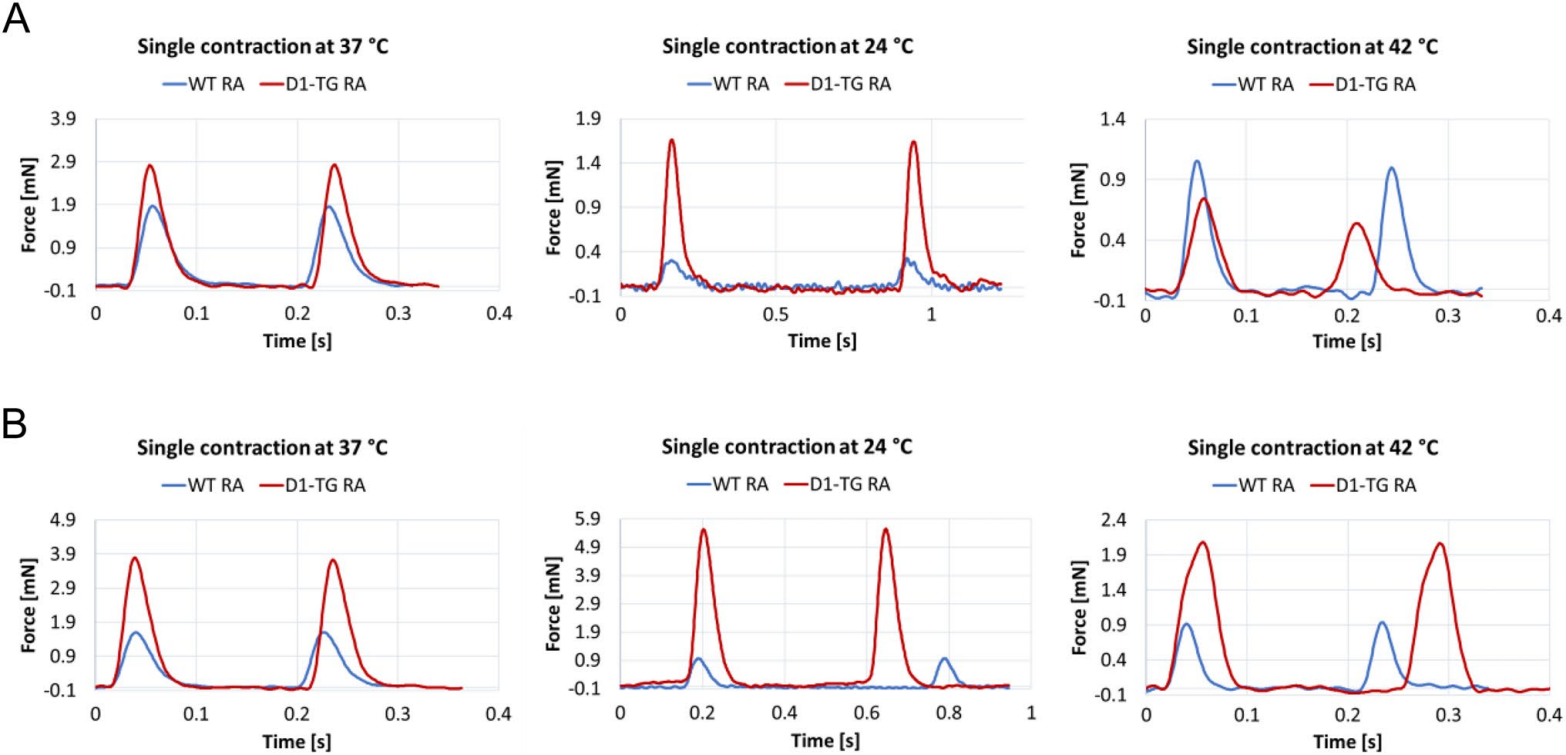

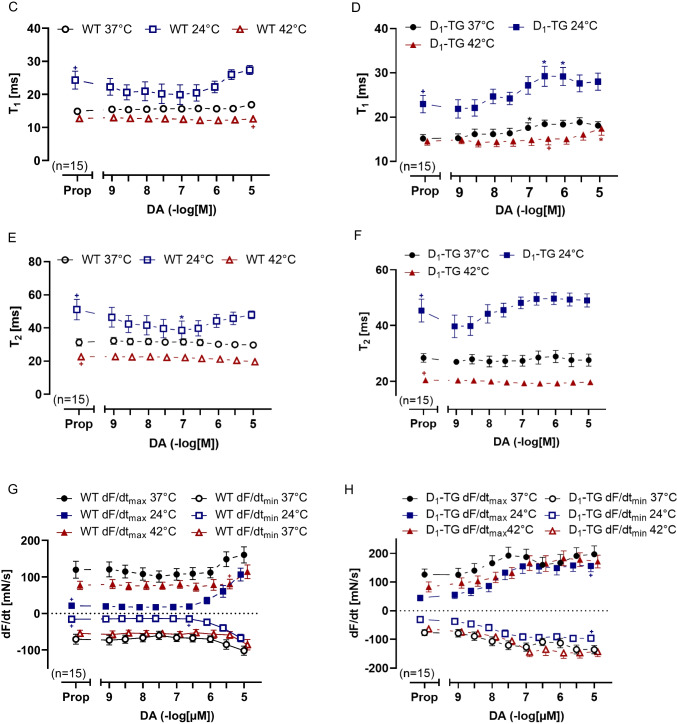
Hyperthermia and hypothermia reduce the effect of dopamine on tension development and relaxation in right atrial preparations, and time to peak tension and relaxation is increased under hypothermia. Original recordings of single contractions under basal conditions (Fig. 7 A), after 300 nM dopamine application (Fig. 7B) and concentration response curves for dopamine in spontaneously beating right atrium (RA) from WT and D_1_-TG under normothermia (37 °C), hypothermia (24 °C) and hyperthermia (42 °C). We added 0.4 µM propranolol to the organ bath in order to block β-adrenoceptors. Dopamine increased time to peak tension in D_1_-TG mice under hypothermal and normothermal conditions but also increased time to relaxation in hypothermia only (**D** and **F**), whereas in WT mice, the peak was reached at higher dopamine concentrations (**C** and **E**). In addition, the effect of dopamine on tension development and relaxation was reduced under hypo- and hyperthermia compared to normothermia (**G** and **H**). Ordinates in **A** and **B** depict developed force of contraction (F in mN), **C** and **D** depict time to tension (T_1_ in ms), **E** and **F** depict time to relaxation (T_2_ in ms), **G** and **H** depict rate of tension development (dF/dt_max_) and rate of tension relaxation (dF/dt_min_) in mN/s. Abscissae in **A** and **B** show time in seconds (s), whereas abscissae in **C** to **H** indicate concentrations of dopamine in negative decadic logarithm of molar concentrations. Temperatures of the organ baths are indicated as circles (37 °C), squares (24 °C), or triangles with tip up (42 °C). “n” indicates the numbers of experiments. * first *p <* 0.05 vs. Prop, ^+^ first *p <* 0.05 vs. 37 °C (ANOVA Bonferroni)

We further studied the left atrium (Fig. 6C and D). Dopamine was more potent to raise the contractility in the left atrium than in the right atrium (Fig. 3E and D). The maximum PIE of dopamine was much higher under hypothermia than under normothermia, whereas the maximum PIE of dopamine under hyperthermia was lower than under normothermia (Fig. 6C versus D). In hyperthermia, dopamine was less potent to elevate the force of contraction in RA than at normothermia (Table 1). This may result at least in part from the negative staircase phenomenon in the mouse atrium. The single contractions in the right atrial preparations during basal conditions at normo-, hypo- and hyperthermia are shown in Fig. 7A, whereas the dopamine effects on a single contraction are shown in Fig. 7B. The time to peak tension and relaxation under basal conditions (Prop) in the right atrial preparations was higher during hypothermia than during normothermia or hyperthermia in WT (Fig. 7C and E) and D_1_-TG (Fig. 7D and F). Dopamine during hypothermia induced a concentration-dependent augmentation in the time to peak tension (Fig. 7B and D) and the time to relaxation (Fig. 7B and F), unlike in the left atrial preparations, where we noted a shortening of the time to peak tension and relaxation (Fig. 5B and D). As in the left atria (Fig. 5B and D), the time to peak tension and relaxation in the right atria during basal control conditions took longer at lower temperatures than at 37 °C and 42 °C. Furthermore, dopamine increased the absolute values of the rate of tension development or relaxation in the right atrial preparations from D_1_-TG mice (Fig. 7H), while in WT, the effect was noticeable at higher dopamine concentrations (Fig. 7G).

**Fig. 8:**
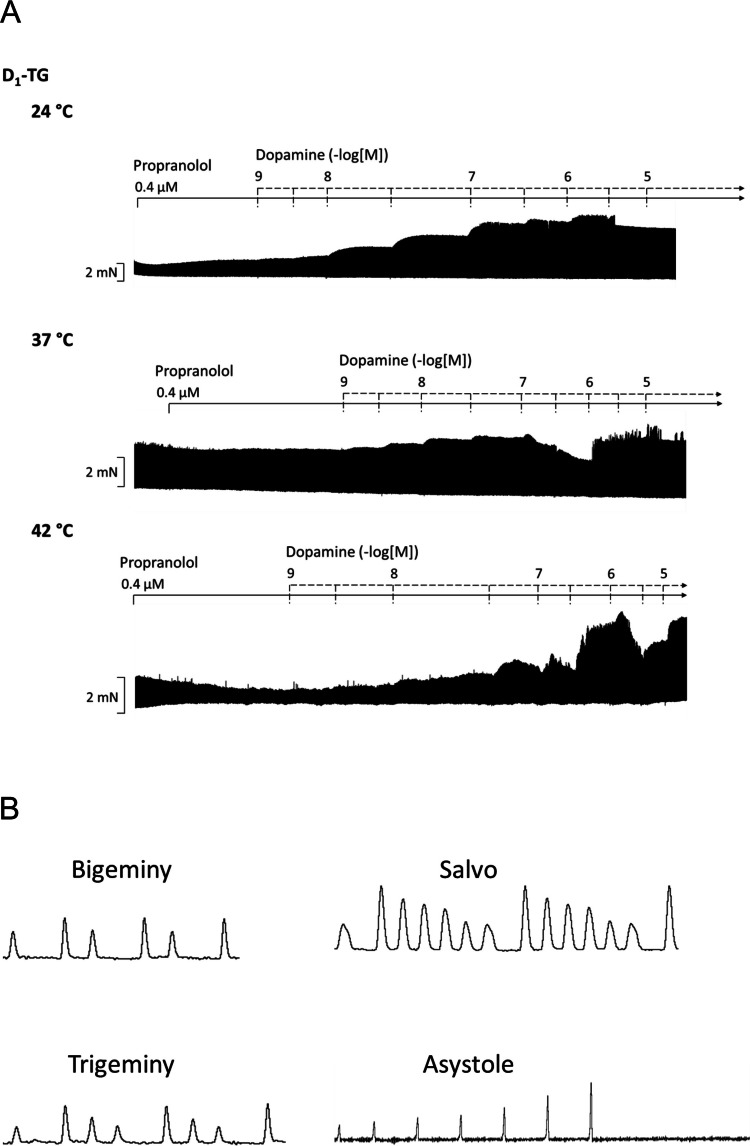

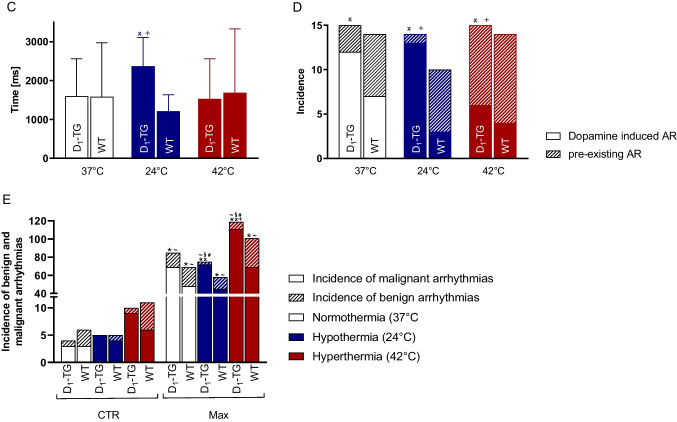
Dopamine induces arrhythmias in WT and D_1_-TG mice under all thermal conditions. Occurrence of arrhythmias at indicated temperatures in right atrial (RA) preparations from WT and D_1_-TG mice. **A** Original recordings of the effect of increasing dopamine concentrations on the force of contractions in RA. We also added 0.4 µM propranolol to the organ bath in order to block β-adrenoceptors. Ordinate depicts developed force of contraction. The abscissa shows increasing logarithmic molar concentrations of dopamine. **B** Original recordings of various malignant arrhythmia types like bigeminy, salvo, trigemini, and asystole. **C** Temperature-dependent average duration of arrhythmias during dopamine addition in WT and D_1_-TG mice. Ordinate depicts the time in milliseconds. ^x^ *p <* 0.05 vs. WT, ^+^ *p <* 0.05 vs. 37 °C. **D** Temperature-dependent incidences of arrhythmias during dopamine addition in WT and D_1_-TG. Ordinate shows the incidences of the arrhythmias. ^x^ *p <* 0.05 vs. WT of dopamine-induced arrhythmias, ^+^ *p <* 0.05 vs. 37 °C of dopamine-induced arrhythmias (2-test). **E** Temperature-dependent average of incidences of benign and malignant arrhythmias before dopamine application (CTR) and during maximal dopamine addition (Max) in WT and D_1_-TG. Ordinate shows the incidences of the arrhythmias. Malignant arrhythmias: * *p <* 0.05 vs. CTR, ^+^ *p <* 0.05 vs. 37 °C, ^x^ *p <* 0.05 vs. WT (2-test). Benign arrhythmias: ^~^ *p <* 0.05 vs. CTR, ^#^ *p <* 0.05 vs. 37 °C, ^§ ^*p <* 0.05 vs. WT (2-test). The abscissae in **C** and **D** show the different temperatures. The number of experiments was *n* = 15

Moreover, we studied whether the incidence and persistence of arrhythmias are changed by the alteration of temperature in D_1_-TG. We display typical original recordings of the right atrium from D_1_-TG in Fig. 8A and original recordings of various malignant arrhythmia types in Fig. 8B. Under hypothermia (24 °C) and normothermia (37 °C), arrhythmias typically developed after higher dopamine concentrations, starting from 100 nM, whereas under hyperthermia (42 °C), arrhythmias were already observed under basal conditions and became more pronounced after higher dopamine induction (Fig. 8A). The results of these experiments are displayed in bar diagrams. It is apparent that, under hypothermia, the arrhythmias lasted longer in D_1_-TG mice than in WT mice (Fig. 8C). In addition, arrhythmias were noted more often under all temperatures in D_1_-TG mice than in WT mice (Fig. 8D). To differentiate between the types of arrhythmias, we have plotted the incidence of benign and malignant arrhythmias (see Methods for our definitions) in another bar diagram (Fig. 8E). It is apparent that in general, malignant arrhythmias occurred more often than benign ones and were higher in D_1_-TG mice than in WT mice (Fig. 8E).

### Human atrial preparations

**Fig. 9:**
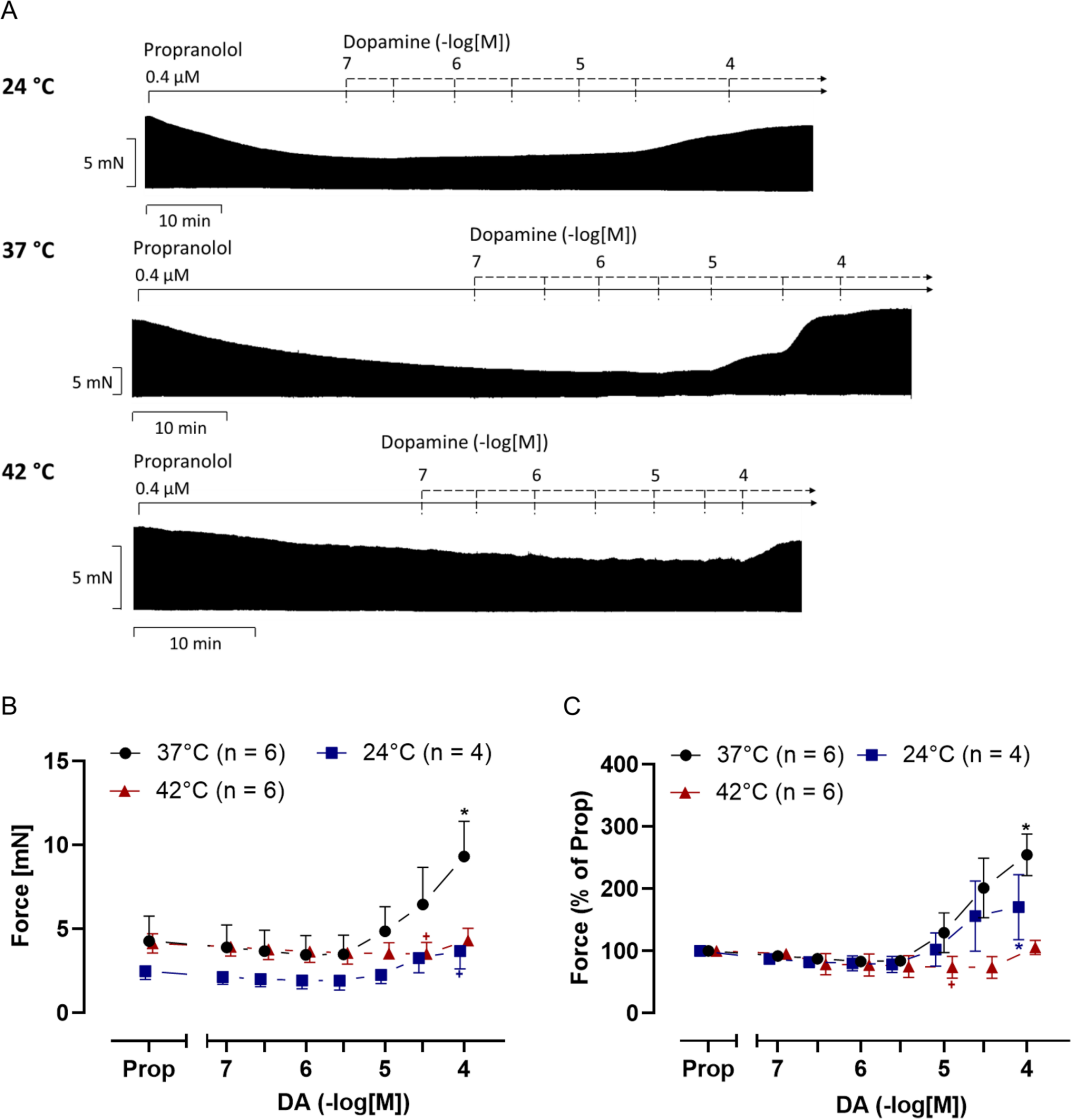
Dopamine increases force of contraction in human atrial preparations (HAP) in a concentration-dependent manner. Original recordings of the effect of increasing concentrations of dopamine in electrically stimulated (1 Hz) human atrial preparations (HAP) under normothermia (37 °C), hypothermia (24 °C) and hyperthermia (42 °C) (**A**). In addition, the concentration-dependent effects of dopamine on force of contraction were summarized in absolute values (**B**) and in % of propranolol (Prop; **C**). In samples, we added 0.4 µM propranolol to the organ bath in order to block β-adrenoceptors. Dopamine increases force of contraction in HAP under all thermal conditions. Ordinates in **A** and **B** depict developed force of contraction in millinewton (mN). Abscissae indicate concentrations of dopamine in negative decadic molar concentrations. Temperatures of the organ baths are indicated as circles (37 °C), squares (24 °C), or triangles with tips up (42 °C). “n” indicates the number of experiments. * first *p <* 0.05 vs. Prop, ^+^ first *p <* 0.05 vs. 37 °C (ANOVA Bonferroni)

In HAPs, dopamine increased force in a time- and concentration-dependent manner. This can be seen in original traces in Fig. 9A. The values on force of contraction (measured in mN) are studied statistically in Fig. 9B. Please note that the PIE of dopamine during normothermia is not seen during either hypothermia or hyperthermia (Fig. 9B and C). Yet, the PIE of dopamine appears during hypothermia at higher concentrations of dopamine (from 10 to 100 µM) and slightly under hyperthermia at the highest dopamine concentration of 100 µM than under normothermia (Fig. 9B and C). Moreover, the maximum augmentation of contractile force is less during hyperthermia than during hypothermia. Thus, high temperatures may diminish the PIE of dopamine more than lower temperatures (Fig. 9B and C).

**Fig. 10:**
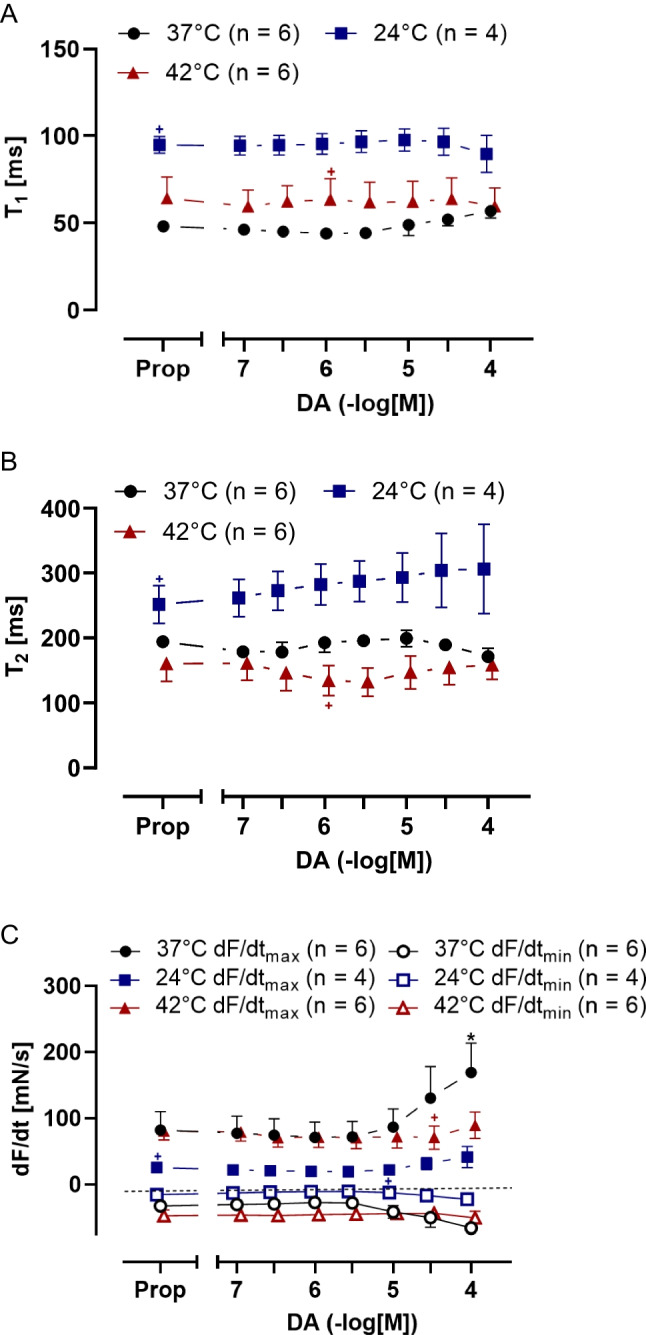
Dopamine increases the rate of tension development in HAP under normothermia and slightly increases relaxation time under hypothermia. Summarized concentration response curves for dopamine in electrically driven (1 Hz) right human atrial preparations. **A** Time to peak tension (T_1_ in ms), **B** relaxation time (T_2_ in ms) and **C** rate of tension development dF/dt_max_ and rate of tension relaxation dF/dt_min_ (in mN/s). Abscissae show negative decadic molar concentrations of dopamine. Temperatures of the organ baths are indicated as squares (37 °C), circles (23 °C), or triangles with tip up (42 °C). “n” indicates the numbers of experiments. * first *p <* 0.05 vs. Prop, ^+^ first *p <* 0.05 vs. 37 °C (ANOVA Bonferroni)

Dopamine concentration-dependently increased the time to relaxation at low and high temperatures (Fig. 10B). Under normothermia, high dopamine concentrations (starting from 10 µM) shortened the absolute values of the time to relaxation (Fig. 10B). It is remarkable that dopamine raised the positive or negative rates of tension development or relaxation under normothermia, beginning at 3 µM. Oppositely, the alterations in the positive or negative rates of tension development or relaxation were less during hyperthermia and lowest during hypothermia (Fig. 10C).

## Discussion

The main new findings here are that temperature alters the potency and efficacy of dopamine to increase force in atrial preparations via human D_1_-dopamine receptors and that the incidence of dopamine to cause arrhythmias is altered by temperature.

We may now compare the present results with dopamine to reports with isoproterenol. Isoproterenol stimulates cardiac β-adrenoceptors. Therefore, one assumes that isoproterenol increases cAMP. Then, cAMP leads in the usual way to an increase in force of contraction and to a an elevation of the beating rate in the mouse or human heart, like dopamine does, in this context (Fig. 1). In left atrial preparations from WT, isoproterenol was less potent to elevate force of contraction at 24 °C than at 37 °C (Gergs et al. 2021). Likewise, the curve for the effect of dopamine on force of contraction in D_1_-TG at 24 °C is dextrally shifted to the curve at 37 °C. Thus, one may draw the conclusion that a similar mechanism may underlie the effects of dopamine and isoproterenol at hypothermia. Also, others found that the concentration-dependent positive inotropic effect of isoproterenol dextrally moved at 25 °C compared to 37 °C: these data were obtained, however, in guinea pig left atrial preparations (Tenner and McNeil 1978, Reinhardt et al. 1978). Our findings in the right atrial preparations were divergent: in the atria from WT, lower temperatures diminished the intrinsic beating rate (Gergs et al. 2021; Hoffmann et al. 2023). However, the positive chronotropic effect of isoproterenol displayed a comparable potency at 24 °C and at 37 °C (Gergs et al. 2021). Similarly, the intrinsic beating rate was less at 24 °C than at 37 °C but was raised in a comparable way at both temperatures in D_1_-TG in the presence of dopamine (Fig. 6B).

In hyperthermia, in guinea-pig left atria, the inotropic effects of isoproterenol were nearly abolished (Reinhardt et al. 1978). Those results compare well with our results in HAP (Fig. 9B) and in left atria from D_1_-TG (Fig. 3E) and right atria from D_1_-TG (Fig. 6D).

Hyperthermia diminished contractility in left atria and elevated the basal rate of contraction in right atrial preparations from D_1_-TG mice. Moreover, the PIE of dopamine was found to be diminished in D_1_-TG mice at hyperthermia.

The temperature dependency of cardiac contractility in general is thought to result from the interplay of the release of Ca^2+^ from the sarcoplasmic reticulum and its uptake into the sarcoplasmic reticulum and the Ca^2+^-sensitivity of the myofilaments (cf. Figure 1). Also in this respect, there are species differences and regional differences. For instance, in trout, at lower (4 °C) and higher (18 °C) than normal (10 °C) temperature, the force of cardiac contraction is regulated by Ca^2+^ entering through the LTCC and released from the SR (Coyne et al. 2000). One explanation is that trout have very little SR as a source of released Ca^2+^ and hence the main way to regulate force is by Ca^2+^ passing into the cardiomyocytes through the LTCC (Coyne et al. 2000). The situation is different in mammals. As concerns regional differences, in the human ventricle (electrically stimulated isolated muscle strips), hypothermia reduces the force of contractions (Hiis et al. 2018). In the human atrium (electrically stimulated isolated muscle strips), hypothermia also reduces the force of contraction (this report, Gergs et al. 2021, Hofmann et al. 2023). This is currently explained by two mechanisms: at low temperature, the Ca^2+^ sensitivity of the myofilaments is reduced, which diminishes the force of contraction. Moreover, there is an inability of SERCA to resequester Ca^2+^ from the cytosol to the sarcoplasmic reticulum (Nakipova et al. 2016). The opposite mechanisms may prevail in hyperthermia, but more research is needed in this regard. There may be an additional role of the LTCC and the inflow of Ca^2+^ into the cell via the sodium-calcium cation exchanger (NCX, Fig. 1). There are some studies that tried to address the cause of temperature-induced changes in the inotropic effect for cAMP-increasing agents. For instance, there are data that β-adrenergic stimulation may reduce the sensitivity of myofilaments to Ca^2+^. Usually, this effect is overcome by an increased release of Ca^2+^ from the sarcoplasmic reticulum (Miyamoto et al. 2001). Such a mechanism may underlie the reduced ability of not only isoprenaline acting via β-adrenoceptors but also dopamine acting here via D_1_-dopamine receptors. D_1_-dopamine receptors and β-adrenoceptors share the ability to raise cAMP levels in the heart. Now it has been reported, in the guinea pig atrium, solely by lowering the temperature, force and the levels of cAMP increased concomitantly (Reinhardt et al. 1978). Interestingly, at 42 °C, basal cAMP levels were lower than control values, and isoprenaline at 42 °C increased less efficiently the force of contraction and cAMP levels, consistent with a causal relationship. In other words, by analogy, we speculate here that dopamine via D_1_-dopamine receptors may increase cAMP (and subsequent biochemical sequelae) more at hypothermia and less at hyperthermia, and this may underlie the temperature-dependent effects of dopamine we noted via D_1_-dopamine receptors in atria from D_1_-TG and HAP.

Moreover, some words may be needed to address the “staircase” or “Treppe” phenomenon or the “Bowditch effect” or the “force-frequency relationship” in the isolated mouse heart. This is an old concept (for history see, e.g. Koch-Weser and Blinks 1963, Puglisi et al. 2011). The basic observation is that simply by increasing the electrical stimulation of a heart preparation that means without adding any drugs like isoprenaline, the force of contraction changes. A positive staircase (“Treppe” in the original German publication on frog hearts: Bowditch 1871) just means as a descriptive term that increased frequency of stimulation leads to increased force generation. The opposite is the negative staircase phenomenon that means an increase in the stimulation rate reduces the force of contraction. The ventricle of the mouse and the healthy human heart shows a positive staircase (mouse: e.g. Stemmer and Akera 1986, Stull et al. 2006; human: e.g. Alpert et al. 1998). In the mouse atrium, a negative staircase is present, whereas in the mouse ventricle, it shows a positive staircase (Stemmer and Akera 1986, Stull et al. 2006, Alpert et al. 1998). The negative staircase in the isolated adult mouse atrium may result from the inability of the mouse atrial sarcoplasmic reticulum to release more Ca^2+^ when the LTCC opens and triggers Ca^2+^ to enter the cells and tries to extrude Ca^2+^ through ryanodine receptors and/or that the sarco(endo)plasmic reticulum Ca^2+^-ATPase (SERCA) is unable to resequester rapidly Ca^2+^ from the vicinity of the myofilaments into the sarcoplasmic reticulum (please see scheme in Fig. 1, cf. Puglisi et al. 2013, Balcazar et al. 2018).

One can ask wherein the clinical relevance of our findings may lie. We would claim that at least our data with human atrial preparations have a clear clinical use. We had shown before that under our experimental conditions, we can detect PIE of dopamine mediated by D_1_-dopamine receptors at 37 °C (Rayo Abella et al. 2024). We confirm these findings at 37 °C. Moreover, at higher temperature (fever) this positive inotropic effect was attenuated (Fig. 9). Hence, one can speculate that in patients with fever, endogenous or exogenous dopamine is unlikely to exert a relevant positive inotropic effect. One can interpret also a drug-induced hyperthermia in this context. For instance, halothane and antipsychotic drugs can induce a malignant hyperthermia which is often combined with an arrhythmia (Gregory and Weant 2021).The same applies to hypothermia. Hypothermia is sometimes used in the clinic to reduce the oxygen demand in muscle in cardiac or brain surgery. Hypothermia is dangerous because it often causes arrhythmias in humans (Dietrichs et al. 2019, Rathjen et al. 2019, Park et al. 2020).

Moreover, our data on arrhythmias are intriguing. We can show that in D_1_-TG, the incidence of atrial arrhythmias is higher than in WT. We interpret this finding as predicting that also in humans, enhanced stimulation of D_1_-dopamine receptors will lead to arrhythmias. This is a plausible chain of events. Stimulation of β-adrenoceptors is accepted to lead to atrial arrhythmias in humans. All cAMP-increasing agents cause arrhythmias more likely in the human heart. D_1_-dopamine receptors increase cAMP levels (Neumann et al. 2023); hence, the same mechanisms (calcium ion overload) can explain the proarrhythmic effects of dopamine and isoprenaline in the human heart. Moreover, it is a well-known clinical problem that infusions of dopamine lead to arrhythmias in intensive care units (De Backer et al. 2010, Zhu et al. 2024). Thence, there is a decline in the clinical use of dopamine to treat low-output cardiac failure (Hiemstra et al. 2019). Our data might speculatively indicate that arrhythmias noted upon dopamine infusion in the clinic are not only mediated by dopamine acting on β-adrenoceptors but in part also are due to dopamine acting on D_1_-dopamine receptors in the heart. This may offer a new way to treat arrhythmias in general: giving also selective D_1_-receptor antagonists.

### Limitations of the study

We have not yet studied the biochemical mechanism(s) underlying the loss of inotropic effect of dopamine at hypo- and hyperthermia. Due to the limited patient samples available to us, we could not study arrhythmogenesis by dopamine in the human atrium. Finally, due to lack of access to the tissue, we have not studied the role of D_1_-dopamine receptors to induce arrhythmias in human ventricular tissue, but this has already been studied in some form by others in living patients at normothermia (Yamaguchi et al. 2020).

In summary, in atrial preparations of D_1_-TG and HAP, dopamine via the D_1_-dopamine receptor exhibited a lower potency and efficacy to elevate the force of contraction in hyperthermia. Dopamine more often induced arrhythmia in D_1_-TG than in WT.

## Data Availability

All source data for this work (or generated in this study) are available upon reasonable request.
